# SurvSig: Harnessing gene expression signatures to uncover heterogeneity in lung neuroendocrine neoplasms

**DOI:** 10.1016/j.csbj.2025.06.010

**Published:** 2025-06-06

**Authors:** Kolos Nemes, Gabriella Mihalekné Fűr, Alexandra Benő, Christopher W. Schultz, Petronella Topolcsányi, Éva Magó, Parth Desai, Nobuyuki Takahashi, Mirit I. Aladjem, William Reinhold, Yves Pommier, Anish Thomas, Lorinc S. Pongor

**Affiliations:** a HCEMM Cancer Genomics and Epigenetics Core Group, Szeged, Hungary; b Doctoral School of Interdisciplinary Medicine, University of Szeged, Szeged, Hungary; c Developmental Therapeutics Branch, Center for Cancer Research, National Cancer Institute, NIH, Bethesda, MD 20892, USA; d Doctoral School of Biology, University of Szeged, Szeged, Hungary; e Genome Integrity and DNA Repair Core Group, HCEMM, Szeged, Hungary; f Department of Medical Oncology, National Cancer Center Hospital East, Kashiwa, Japan

**Keywords:** Lung neuroendocrine, Expression signature, Stratification, Clustering, Survival, Machine learning

## Abstract

The advances in the field of cancer genomics have enabled researchers and clinicians to identify altered pathways and regulatory networks that differentiate subtypes manifesting as differential phenotypes of lung neuroendocrine neoplasms (NENs). The clinical heterogeneity observed among lung NEN subtypes reflects underlying biological distinctions, including differential mutation patterns, epigenetic changes and immune microenvironment activities. Although in many cases only a handful of underlying genes are used to differentiate patients, broader gene signatures might result in finer separation and help identify patients with differential survival. Lung NENs are vastly underrepresented in pan-cancer studies, resulting in lacking options to explore datasets. To this end, we developed a freely available website (https://survsig.hcemm.eu/) which allows users to upload potential genes of interest, perform patient clustering, compare survival and explore gene expression signature of lung NENs. Leveraging these biological differences enhances the accuracy of gene expression-based prognostic classifiers like SurvSig.

## 1 Introduction

Lung neuroendocrine neoplasms (NENs) account for approximately 20–25 % of all lung cancers and exhibit a wide range of clinical behaviors, from indolent to highly aggressive forms [1]. Lung NENs are classified into lung neuroendocrine tumors (NETs), representing mainly the well differentiated carcinoids (CARCI), and the poorly differentiated lung neuroendocrine carcinomas, encompassing large cell neuroendocrine carcinoma (LCNEC) and small cell lung cancer (SCLC) [2]. Carcinoids can be further separated to low-grade typical carcinoids and intermediate-grade atypical carcinoids, having relatively better prognosis compared to NECs. In contrast LCNEC and SCLC are high-grade and poorly differentiated tumors associated with aggressive behavior, rapid progression, and poor survival outcomes.

The molecular pathogenesis of lung NENs is complex and varies across the different subtypes, typically lacking common oncogenic driver mutations found in non-small cell lung cancer (NSCLC), such as *KRAS* and *EGFR* in lung adenocarcinomas [3]. Carcinoid tumors present with lower mutational burdens, often harboring somatic mutations in chromatin remodeling genes, such as *MEN1* and *ATRX* [4], [5]. On the other hand, SCLC and LCNEC are characterized by high mutational burdens and frequent alterations in key tumor suppressor genes, such as the *TP53* gene. In addition, *RB1* gene mutations can be found in most SCLC tumors, and approximately half of LCNEC tumors. In case of LCNEC tumors with no *RB1* mutations are often enriched with *STK11* and *KEAP1* gene mutations, potentially reflecting distinct subgroups [6], [7].

Recent advances have focused on stratifying lung NENs based on transcriptional profiles, resulting in the classification of patients into distinct molecular subtypes. Carcinoid tumors have been divided into three major molecular subtypes [5], [8]: “cluster A1” is defined by *DLL3* and *ASCL1* expression, “cluster A2” manifests with SLIT/ROBO pathway downregulation, and “cluster B” is comprised mainly of atypical carcinoids and tumors with *MEN1* mutations which display poorer outcomes [5]. A fourth emerging subtype, known as supra-carcinoids, has also been identified and shows molecular similarities to high-grade tumors [5]. LCNEC tumors have also been subdivided into two primary categories: type I, which are highly neuroendocrine and enriched for *STK11* and *KEAP1* mutations, and type II, which exhibit less neuroendocrine features, including enrichment of *RB1* mutations and upregulation of the NOTCH pathway [7]. SCLC, meanwhile, can be categorized into molecular subtypes based on the expression of lineage-specific transcription factors *ASCL1*, *NEUROD1*, *POU2F3*, and *YAP1* [9]. Taken together, these analyses highlight that lung NENs comprise diverse subtypes exhibiting distinct biological behaviors beyond traditional morphological classifications. Consequently, identifying novel patient subgroups, potentially independent of established molecular clusters, may lead to improved prognostication, refined therapeutic strategies, and ultimately better clinical outcomes.

As genome-wide profiling gains popularity, the identification of transcriptional signatures associated with various conditions and cellular states has become increasingly common. These signatures have proven useful for identifying patient subtypes and stratifying cell lines, demonstrating that complex gene expression patterns can provide more precise classification of patient groups than single-gene analyses [10], [11], [12], [13], [14]. By integrating multi-omics data, these have led to identification of dysregulated pathways, such as p53 and ATM in neuroendocrine neoplasms, related to miRNA-mediated regulation with the potential for use as prognostic markers [15]. Furthermore, these signatures can have therapeutic implications, where a notable example is the identification of the inflamed subtype in SCLC, which has significantly better response to different treatments, including immunotherapy [12], [16], [17].

Unfortunately, lung NENs are often underrepresented in large-scale pan-cancer studies, such as The Cancer Genome Atlas (TCGA), due to challenges associated with obtaining sufficient tumor specimens. Consequently, the availability of tools for exploring lung NEN data is extremely limited, impeding both research advancements and clinical interpretations. Our aim was to address these limitations by developing an online tool that that enables the interactive exploration of these datasets. For this reason, we have compiled publicly accessible gene expression data from multiple lung NEN cohorts and developed a freely available online website allowing users to upload and interactively analyze gene signatures with various machine learning approaches. This novel and unique approach will facilitate identifying potential subgroups in these heterogeneous diseases, discovery potential biomarkers and aiding in the interpretation of complex transcriptional data.

## 2 Results

**Fig. 1:**
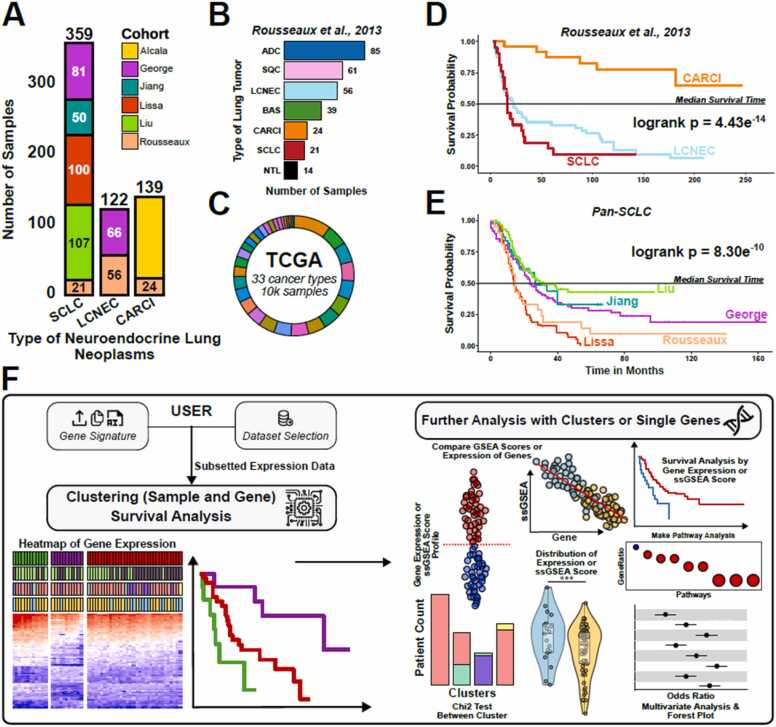
Datasets and SurvSig website. A) number of samples from each dataset for the three lung Nen histologies. B) number of patients for each histology of the rousseaux cohort. C) summary of the TCGA integrated dataset. D) Kaplan-Meier survival plot of lung histologies in the rousseaux cohort. E) Kaplan-Meier survival comparison of SCLC patients from the 5 collected cohorts. F) summary of the SurvSig website.

We collected publicly available gene expression data for over 600 lung NENs across six datasets, encompassing the three major histological subtypes (Fig. 1**A**). The majority of samples are from small cell lung cancer (SCLC, n = 359) [6], [18], [19], [20], [21], which is the most prevalent NEN subtype, followed by carcinoid tumors (n = 139) [4], [5], [21] and large cell neuroendocrine carcinoma (LCNEC, n = 122) [7], [21]. Among the datasets, one cohort includes transcriptional data for non-NEN tumors, allowing for cross-histology comparisons (Fig. 1**B**) [21]. Additionally, we incorporated the TCGA (The Cancer Genome Atlas) integrated cohort (33 cancer types, >10k samples) [22], enabling the examination of transcriptional patterns across other cancer types (Fig. 1**C**).

Patient survival varies significantly among the main histological subtypes of lung NENs, demonstrating a pronounced bimodal distribution. Carcinoid tumors are associated with notably favorable outcomes, where median survival was not reached in either the *Alcala* or *Rousseaux* cohorts. High-grade SCLC and LCNEC display poor survival outcomes, with median survivals of approximately 15 and 20 months, respectively (*Rousseaux* cohort, Fig. 1**D**). Detailed survival analysis of SCLC patients expectedly found significantly better prognosis in limited-stage and chemo-naive cohorts (George-SCLC, Liu-SCLC and Jiang-SCLC) compared to the *Lissa* and Rousseaux cohorts, where most patients received systemic chemotherapies (Fig. 1**E**). Comparisons between LCNEC and carcinoid tumors across the datasets showed no significant differences in survival (Fig. S1A**-B**).

To support data mining and exploration of gene expression signatures, we developed the ***SurvSig*** website (https://survsig.hcemm.eu/), which facilitates clustering-based analysis of complex gene expression patterns (Fig. 1**F**). After uploading a gene signature or gene list (up to 2000 genes), the website performs dimensionality reduction and clustering to group patients based on similar transcriptional profiles. Multiple dimensionality reduction and clustering algorithms are available and customizable by the user. The generated clustered expression heatmaps allow for survival comparisons among patient clusters, revealing potential differences in survival outcomes. Multivariate analysis and patient annotation enrichment can also be compared between patient clusters to account for clinical variables. Additionally, uploaded genes can be clustered to identify groups with similar expression patterns. ***SurvSig*** also includes gene set enrichment analysis for both uploaded and clustered gene sets to quantify pathway activity, which can be used for survival analysis in a single-gene context. The expression profiles of single genes and enrichment scores can be compared through correlation and violin plots, where samples can be grouped and color coded based on annotations and clinical characteristics.

### 2.1 Neuroendocrine gene signatures

**Fig. 2:**
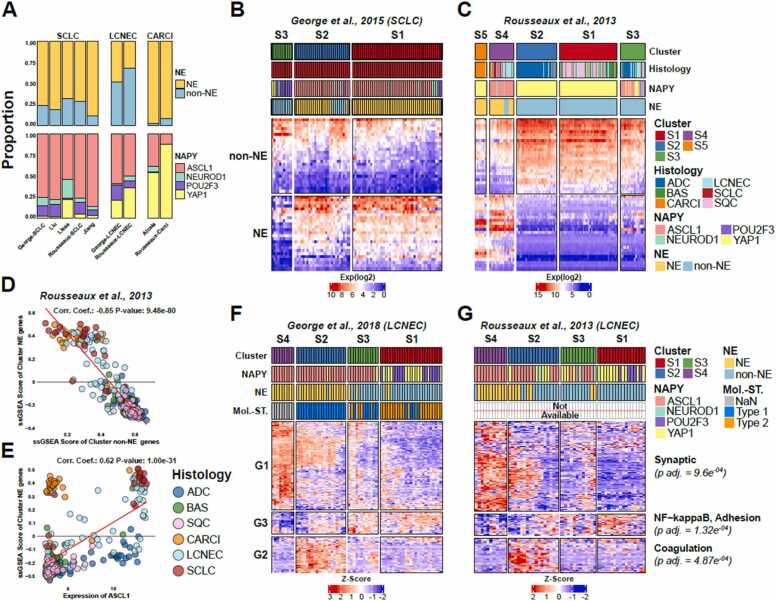
Transcriptional patterns defining lung NENs. A) distribution of rudin subtypes (NAPY, top) and NE subtypes (NE50 signature, bottom) of lung NENs. B) expression heatmap of the NE50 gene signature using the George-SCLC cohort (RNA-seq). C) expression heatmap of the NE50 gene signature using the rousseaux cohort (microarray). D) spearman correlation of NE and non-NE geneset activities defined in the NE50 signature using the rousseaux cohort. E) correlation of ASCL1 expression and NE geneset activity (from NE50 signature) using the rousseaux cohort. F-G) expression heatmap of genes that defined molecular subtypes [7] using the F) George-SCLC cohort (RNA-seq) and G) LCNEC tumors from the rousseaux cohort (microarray).

We classified tumors into neuroendocrine (NE) and non-neuroendocrine (non-NE) subtypes using the NE50 gene signature [23], which includes 25 genes highly expressed in NE tumors and 25 genes highly expressed in non-NE SCLC tumors (Fig. 2**A,** Table S1). A representative heatmap for the signature in the *George-SCLC* cohort can be seen in Fig. 2**B**. Approximately 75–80 % of SCLC tumor cases scored positively for NE characteristics. Conversely, a substantial proportion of LCNEC tumors exhibited stronger non-NE features (50 % in the *George-LCNEC* and 70 % in the *Rousseaux-LCNEC* cohorts). Carcinoid tumors were predominantly highly neuroendocrine, except for a few cases that were enriched for non-NE genes, potentially linked to the emerging supra-carcinoid subtype (Fig. S2A**-B**).

Next, each patient from all lung NEN datasets was classified using the Rudin classification for SCLC tumors [9], which assigns subtypes based on the expression of *ASCL1* (A), *NEUROD1* (N), *POU2F3* (P), or *YAP1* (Y). As expected, chemo-naive SCLC cohorts were predominantly enriched for *ASCL1*+ tumors (*George-SCLC, Liu* and *Jiang* cohorts), whereas the highly treated *Lissa* cohort had higher prevalence of *YAP1*+ tumors (Fig. 2**A**). LCNEC tumors presented a significantly greater proportion of *YAP1*+ cases compared to SCLC. The most notable difference was seen in carcinoid tumors, where the tumors in the *Alcala* cohort had significantly fewer YAP1 + cases compared to the carcinoid tumors in the *Rousseaux* cohort despite retaining similar NE signature characteristics.

While most lung NENs retain distinct neuroendocrine (NE) characteristics, a subset exhibits prominent non-NE features, showing molecular similarities to non-small cell lung cancers (NSCLC) (Fig. 2**C**). This observation is supported by ssGSEA-based enrichment analyses, which demonstrate strong negative correlations between NE and non-NE gene expression across all lung tumor histologies (Fig. 2**D**, with differential activity quantified in Fig. S2C). Notably, while ASCL1—a key NE marker—predictably shows positive correlation with NE-specific genes in the NE50 gene set, its expression is absent in many carcinoids (Fig. 2**E**, with sample tumor histology indicated by color). Moreover, some NSCLC adenocarcinomas (ADC) exhibit high *ASCL1* expression while maintaining a non-NE phenotype, as observed in both the *Rousseaux* cohort and *TCGA* datasets (Fig. S2D**-E**). These findings reinforce the notion that although *ASCL1* serves as a critical regulator in neuroendocrine cells, additional factors are necessary to drive full neuroendocrine differentiation ^24^.

We subsequently assessed the gene signature employed to classify the Type I, Type II, and ‘nan’ (unassigned or unknown) molecular subtypes within the George-LCNEC tumors cohort (clustered data available in Table S2) ^7^. Using two-dimensional UMAP dimensionality reduction combined with dynamicTreeCut clustering, we identified four distinct patient groups and three primary gene sets (Fig. 2**F**). This clustering analysis delineated the George-LCNEC cohort into four groups that correlated with molecular subtypes: S1 was predominantly Type II, S2 exclusively Type I, S3 a mixture of Type I and II, and S4 exclusively ‘nan’. The S1 cluster was characterized by POU2F3 + and YAP1 + tumors, while clusters S2-S4 were nearly exclusively ASCL1 + . This pattern was also reflected in the NE scores, with S1 displaying a non-NE profile, S2 and S4 being mainly NE, and the mixed S3 cluster showing non-NE characteristics despite elevated ASCL1 expression.

The identified clusters were differentially enriched in three gene sets (G1-G3, gene ontoloy in Table S3). G1 was associated with the ‘nan’ subtype and enriched in synaptic pathway-related genes, showing significantly higher expression of neuroendocrine markers such as INSM1 and SYP (Fig. S2E). G2 was linked to the Type I subtype, with enrichment in coagulation pathways, while G3 corresponded to the Type II subtype, mainly composed of non-NE tumors and enriched for the G3 gene cluster, which includes genes related to NFKB and adhesion pathways. Notably, we observed similar clustering patterns within LCNEC samples from the Rousseaux cohort (Fig. 2**G,** distribution of INSM1 and SYP expression in Fig. 2**F**), where expression was quantified using microarray in comparison the RNA-seq based Alcala cohort, underscoring the robust reproducibility of this gene signature in the molecular classification of LCNEC tumors.

### 2.2 Identifying signatures related to patient annotations

We implemented the option to identify genes of interest through various statistical approaches, enabling the detailed exploration of the datasets (“Gene Set Finder” tab on ***SurvSig***). This can be achieved either by using an entire patient cohort, or a subset of patients, considering either the full gene set or a gene list uploaded by the user. Two main approaches are implemented: 1) selecting informative genes based on expression profiles, such as standard deviation, PCA, SVD and other approaches; 2) identifying gene groups with differential expression profiles between predefined patient groups, such as clinical characteristics or annotations.

**Fig. 3:**
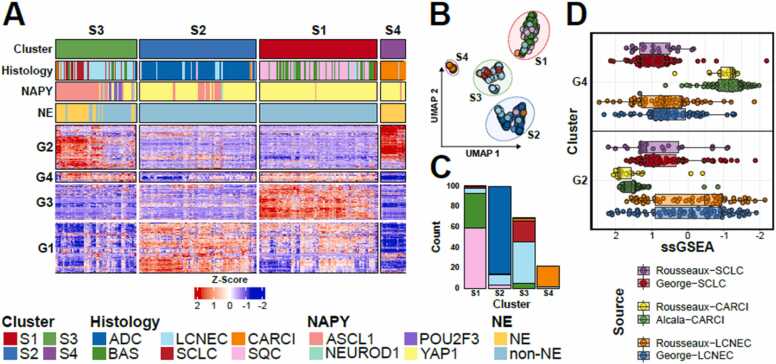
Variable genes across lung tumor histologies. A) expression heatmap of 500 most variable genes identified in the rousseaux cohort. B) UMAP representation and clustering of patient samples. C) histology composition of the identified patient clusters. D) normalized enrichment scores of NE specific genesets from multiple data sources.

To illustrate this feature, we extracted the top 500 most variable genes in the mixed *Rousseaux* based on standard deviation (Fig. 3**A,** Table S4) including six lung cancer histologies (SCLC, LCNEC, CARCI, SQC (Squamous cell carcinoma), ADC (Adenocarcinoma) and BAS (Basaloid) histologies). Cluster analysis and heatmap representation of patients highlighted four distinct groups with distinct behaviors, defined by UMAP dimensionality reduction and dynamicTreeCut clustering (Fig. 3**B**). The S1 cluster comprised SQC and BAS (a rare subtype of SQC) tumors, while the S2 cluster predominantly included ADC tumors. Most SCLC and LCNEC tumors clustered together in the S3 group, independent of NE status, whereas nearly all carcinoid tumors were grouped in the S4 cluster (Fig. 3**C**).

The gene clusters active in lung NEN tumors (G2) were highly enriched for neuronal and secretory pathways (Fig. S3A**,** gene ontology results can be found in Table S5). This gene cluster was highly expressed in all three lung NEN histologies (SCLC, LCNEC and CARCI). In contrast, the G4 gene cluster was more inactive in carcinoid tumors compared to high-grade lung NENs, enriched for replication related pathways (Fig. S3B**,** Table S5). This differential activity was reinforced by comparing normalized enrichment scores among the lung NEN datasets (Fig. 3**D** and Fig. S3C), where the G2 cluster was more active in carcinoid tumors, followed by SCLC tumors and finally LCNEC tumors. In contrast the G4 cluster was more active in SCLC and LCNEC tumors compared to the low-grade carcinoids.

### 2.3 Molecular and prognostic signatures in carcinoid tumors

Carcinoid tumors can be categorized to typical (TC) and atypical (AC) cases, that have recently been further classified into 4 molecular subtypes (termed A1, A2, B and supra-carcinoids) using distinct gene expression signatures [5]. The three gene signatures (A1 vs A2, A1 vs B and A2 vs B signatures [5]) yielded reproducible results using the original *Alcala* cohort, highlighting the transcriptional differences among the molecular subtypes (Fig. S4A), also seen in the carcinoid tumors from the *Rousseaux* cohort (Fig. S4B).

**Fig. 4:**
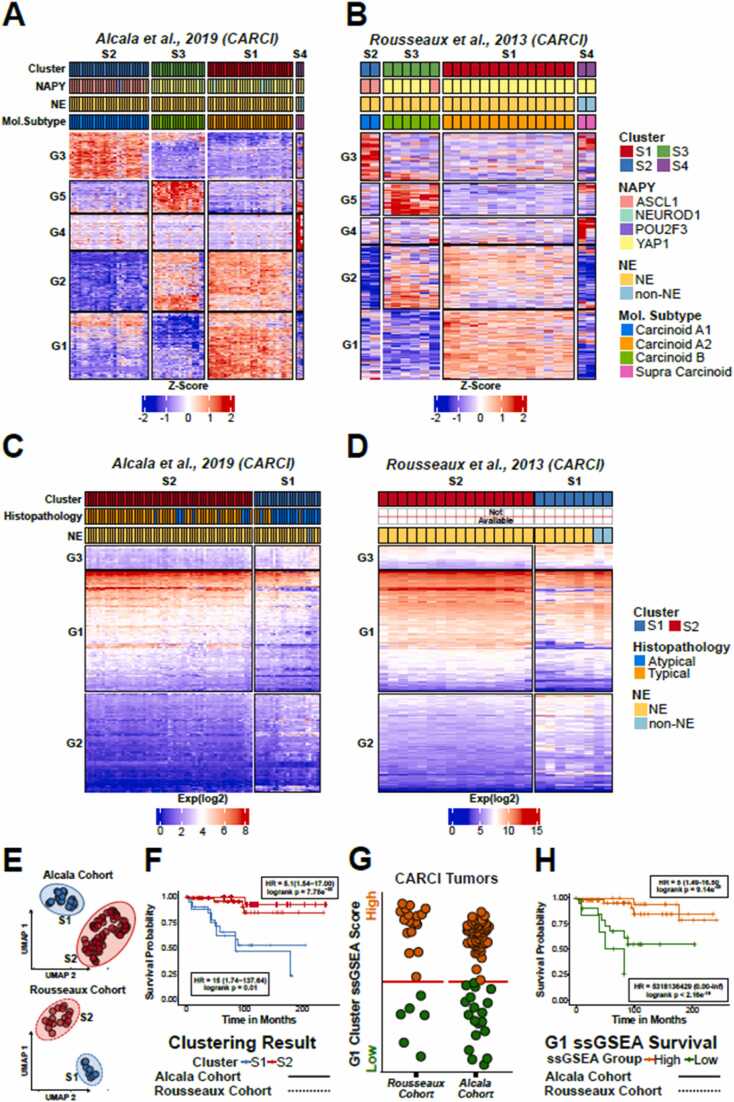
Expression patterns of carcinoid tumors. A-b) clustering of 1k genes identified by artificial neural network defining molecular subtypes of carcinoids in the A) alcala and B) rousseaux cohorts. C-d) clustering of patients using 358 genes identified by the nearest centroids classifier that differentiate TC and AC carcinoids using the C) alcala (RNA-seq based expression) and D) rousseaux (microarray-based expression) cohorts. E) UMAP and clustering of alcala (top) and rousseaux (bottom) cohorts. F) Kaplan-Meier survival comparison of patient cohorts from the two carcinoid cohorts. G) ssGSEA activity and cutoffs identified with automatic cutoff selection of the G1 gene list enriched in TC. H) Kaplan-Meier survival plots of high and low G1 gene set activities.

Using ***SurvSig***, we constructed a single gene signature that can differentiate the molecular histologies, without the need of multiple gene lists for classification. For this, we identified 1k genes that describe the four molecular subtypes using an artificial neural network (MLP model from SciKit-learn package in python) implemented on ***SurvSig*** (Table S6**)**. Patient clustering using the newly defined genes by PCA dimensionality reduction (with whitening enabled) and dynamicTreeCut algorithm (set to complete linkage method) separated the *Alcala* carcinoid tumors completely based on the predefined molecular subtypes (Fig. 4**A**). The A1 tumors were enriched for neuronal pathways (G3 genes), the A2 tumors were enriched for metal ion stress response (G1 genes) and wound healing pathways (G2 genes), the B tumors were enriched with synaptic pathways (G5 genes) and wound healing (G2 genes), while the supra-carcinoids were enriched for protein kinase and serine endopeptidase pathways (G4 genes) (gene ontology can be found in Fig. S4C and Table S7). Using the gene signature, we were able to annotate the molecular clusters of the carcinoid tumors from the *Rousseaux* cohort (microarray-based expression quantification) as well, which yielded very similar expression heatmaps despite difference in expression quantification technologies (Fig. 4**B**).

To better differentiate gene expression patterns among the TC and AC subtypes, we performed a grid search using nearest centroids classifier (see Methods section), which identified 358 genes that are differentially enriched in TC and AC carcinoids from the *Alcala* cohort (Table S8). Cluster analysis of the gene signature identified three groups of genes, of which two had marked differential activity between the two subtypes (Fig. 4**C**). The G1 gene set was more active in typical carcinoids and were enriched for GTP pathways, while the gene set enriched in atypical carcinoids were enriched for spindle and mitotic pathways (Fig. S5A). Importantly, we observed similar separation in the independent carcinoid samples from the *Rousseaux* mixed cohort, highlighting the reproducibility of the gene set (Fig. 4**D**).

As expected, the patient cluster that was enriched for typical carcinoid signatures had significantly better survival, than the patient cluster enriched for atypical signatures, using the default UMAP dimensionality reduction and clustering patients to two groups using k-means (excluding samples that were included from another study with no clinical information with the “LC exclusion” toggle, and turning off Z-scoring of data). This trend was visible using both the *Alcala* cohort, and carcinoid tumors from the *Rousseaux* cohort (Fig. 4**E-F)**. We also tested whether gene set enrichment quantified by ssGSEA (Single Sample Gene Set Enrichment Analysis) of the two gene signatures (G1 and G2) could be used as surrogate markers to predict survival. The analysis reinforced the observation that patients displaying higher activity of the typical carcinoids G1 genes had significantly better outcomes in both datasets (Fig. 4**G-H**). In contrast, patients with signature enrichment of the G2 genes, enriched in atypical carcinoids, had significantly worse outcomes (Fig. S5B). The significance was retained even when correcting the p-value for multiple testing from the automatic cutoff selection of all ssGSEA based survival analyses (Fig. S5C).

## 3 Discussion

In this study, we compiled a comprehensive collection of publicly available gene expression data from over 600 lung neuroendocrine neoplasms (NENs), encompassing carcinoids, large cell neuroendocrine carcinoma (LCNEC), and small cell lung cancer (SCLC). By integrating this data into an interactive online platform called ***SurvSig***, we enabled the analysis of gene expression signatures using various machine learning approaches. Our findings demonstrate that complex transcriptional patterns can effectively classify lung NEN subtypes and are associated with distinct clinical outcomes, particularly in differentiating typical and atypical carcinoid tumors.

The application of transcriptional profiling allowed us to classify tumors based on established molecular signatures, such as the Rudin classification for SCLC and the NE50 gene signature for neuroendocrine differentiation. We observed that carcinoid tumors exhibit diverse molecular subtypes with distinct gene expression patterns, which correlate with patient survival. Specifically, we identified gene clusters that differentiate typical carcinoids, associated with favorable prognosis, from atypical carcinoids, which have poorer outcomes. These gene signatures were consistent across independent cohorts, highlighting their robustness and potential utility in clinical settings.

Lung NENs have distinct pathogenic mechanisms related to their development, resulting in a heterogeneous group with distinct pathogenetic profiles across their subtypes. A recognized premalignant condition, diffuse idiopathic pulmonary neuroendocrine cell hyperplasia (DIPNECH), has been implicated in the early development of typical and atypical carcinoids, particularly among non-smoker females. Environmental factors, most notably nicotine and hypoxia related to smoking, contribute significantly to the pathogenesis of high-grade tumors. Notably, the mutational burden is substantially higher in SCLC than in carcinoids, reflecting their more aggressive behavior and accumulation of genetic alterations over time. Although smoking is a major risk factor for NECs, its role in carcinoid development remains unclear, suggesting distinct etiologies within the lung NEN spectrum [25].

Our work builds upon previous studies that have attempted to stratify lung NENs using molecular characteristics. By leveraging machine learning algorithms within ***SurvSig***, we were able to refine these classifications and identify novel gene signatures that may serve as prognostic biomarkers. The ability to reproduce these findings across multiple datasets underscores the importance of integrating large-scale genomic data to enhance our understanding of tumor biology and improve patient stratification. Using SurvSig, we were able to construct gene signatures that stratify patients into molecular clusters using a single list. Importantly, many identified genes have been recently validated to be discriminators of these clusters using protein expression through immunohistochemistry [26], [27]. Notable examples are enrichment of OTP in A1 and A2 carcinoids, ASCL1 in A1, HNF1A in A2 and B clusters and ANGPTL3 in B clusters (Fig. 4A, Table S6).

The distinct clinical trajectories observed in lung NENs can be directly attributed to their underlying biological diversity. Typical and atypical carcinoids often harbor mutations in the MEN1 gene and exhibit low proliferation rates, which correlate with their generally indolent clinical behavior. Conversely, high-grade neuroendocrine carcinomas—such as SCLC and LCNEC—are characterized by frequent genetic alterations in TP53 and RB1, extensive genomic instability, and aggressive proliferation. Recent literature also highlights the importance of tumor plasticity, with documented instances of non-small cell lung cancer (NSCLC) undergoing histological transdifferentiation into SCLC following targeted therapy resistance [28], [29]. Furthermore, a recent study has demonstrated the ability of carcinoids to differentiate to high-grade tumors by acquiring amplifications of cell-cycle regulating genes through chromothripsis [30].

Additionally, the immune microenvironment markedly varies among subtypes, with carcinoids typically displaying low immune infiltration and an immunologically 'cold' phenotype [31], [32], whereas SCLC and LCNEC frequently present a heterogeneous immune contexture, including immunosuppressive environments that diminish response to immunotherapeutic strategies [12], [32], [33], [34]. These biological differences are critical for interpreting the transcriptomic signatures captured by SurvSig, underpinning its utility as both a prognostic and biologically informative tool. Future studies integrating genomic, epigenomic, and immune profiling could further refine our understanding of these differences, potentially improving patient stratification and guiding subtype-specific treatment approaches.

Our study has several limitations to take into account. SurvSig relies solely on transcriptional patterns for sample stratification and does not incorporate genomic features such as mutations or copy number variations (CNVs) [4], [5], [6], [7], [35]. These genetic alterations are known to influence gene expression and can act as confounding factors, as demonstrated in carcinoid tumors (e.g., MEN1 mutations) and LCNEC. However, previous studies have also shown that transcriptional signatures associated with these alterations are detectable in tumors lacking the corresponding mutations, underscoring the role of alternative mechanisms—such as epigenetic inactivation [24], [36]—that can lead to similar phenotypic outcomes. Additionally, given the reliance on publicly available datasets, cohort selection bias remains a potential limitation, particularly in terms of sample diversity and completeness of clinical annotation. Moreover, it is well-established that comparing RNA expression levels directly with protein abundance has inherent limitations.

While SurvSig was specifically designed and validated for lung neuroendocrine neoplasms, the underlying methodology is broadly applicable and could, in principle, be extended to other neuroendocrine malignancies such as pancreatic neuroendocrine tumors (PanNETs) [37], [38]. Given the shared neuroendocrine transcriptional programs across tissue types, such an extension is technically feasible, as previous research has demonstrated common transcriptional and epigenetic programs in other neuroendocrine neoplasms [37], [39]. However, systematically evaluating its performance in non-pulmonary neuroendocrine cancers requires tumor-specific reference cohorts with well-annotated survival data, which falls beyond the scope of the current study. Future work may explore this direction as suitable datasets become available.

In summary, the ***SurvSig*** platform offers a versatile tool for the exploration of gene expression signatures in lung NENs. By facilitating biomarker discovery and enabling the interpretation of complex transcriptional data, ***SurvSig*** can aid in the development of personalized therapeutic strategies. SurvSig has the potential to uncover novel transcriptional signatures and genes, aiding the identification of candidate markers capable of differentiating lung NET histologies; however, these findings require further validations in the clinical setting. Continued development of such tools are essential for translating genomic insights into actionable clinical interventions, ultimately improving patient outcomes in lung NENs.

## 4 Material and methods

### 4.1 Data collection

Processed gene expression and survival data were collected from multiple publicly available neuroendocrine lung tumor cohorts. For small cell lung cancer (SCLC), data were sourced from George-SCLC [6] through cBioPortal [40], Lissa-SCLC [18], Jiang-SCLC [19], and Liu-SCLC [20], covering both treatment-naive and metastatic cases. Large cell neuroendocrine carcinoma (LCNEC) data were obtained from George-LCNEC [7]. Pulmonary carcinoid tumor data were derived from Fernandez-CARCI [4] and Alcala-CARCI [5]. The Cancer Genome Atlas (TCGA) integrated cohort was also included [22], covering 33 different cancer types for comparative analysis, selecting earlier sample time points for duplicate entries. The Rousseaux mixed lung tumor cohort [21] was also included, where expression was profiled using microarrays. Raw cel expression files were obtained from GEO, imported with the *read.affybatch* function from the *affy* package [41], followed by normalization with the *rma* function*.* Gene expression scores were obtained with the *jscores* function from the JetSet package [42]. Gene names across all cohorts were standardized to HUGO symbols through an automated mapping process to correct outdated or non-standard gene names. Unmapped genes were excluded, and duplicates were removed by retaining the cases with highest mean expression. Datasets where expression was summarized in normalized read counts were log2-transformed to stabilize variance. For all cohorts except TCGA, NAPY [9], neuroendocrine (NE) [23], and epithelial-mesenchymal transition (EMT) scores [43] were calculated to assess tumor characteristics. These scoring methods were applied uniformly across the cohorts, ensuring consistent analysis across datasets. Cohorts are represented as individual sets on the SurvSig website, which can be selected under the “Select a Dataset” drop-down menu.

### 4.2 SurvSig website implementation

Python and R were used for data analysis and visualization. A web application developed using Python and Streamlit (1.39) was deployed on a Linux Debian (6.1.38) server, with integration of R for additional statistical analysis. Python (3.12) and R (4.3.3) were used for consistent analysis. A brief example about using SurvSig can be found on the landing page, as well as under the “Help” tab, where several short introduction videos can be found related to the different functionalities.

### 4.3 Dimensionality reduction and clustering

Dimensionality reduction methods included Uniform Manifold Approximation and Projection (UMAP), t-distributed Stochastic Neighbor Embedding (t-SNE), Principal Component Analysis (PCA), and Multidimensional Scaling (MDS). UMAP [44] was implemented using umap-learn (0.5.6), while t-SNE [45], PCA, and MDS were applied using scikit-learn (1.5.1 and 1.5.2). Non-negative Matrix Factorization (NMF), implemented with scikit-learn and bignmf (1.0.5) [46], was also performed for clustering purposes. PHATE, used for nonlinear dimensionality reduction, was implemented with phate (1.011) [47]. On the SurvSig website, we implemented several advanced settings that users can modify for each method. In case of UMAP, users can modify minimal distance, number of neighbors and distance metric. For t-SNE, users can modify perplexity, number of iterations, distance metric and embedding method. In PCA analysis users can modify tolerance, whitening and SVD solver. In MDS, users can select number of iterations, epsilon value and use of metric MDS. In case of NMF analysis, in the standard NMF, users can select iterations, tolerance, method of initialization, beta loss, numeric solver and randomization of order of coordinates, while in the NMF clustering option, users can select number of iterations, number of trials and Lamb’s value. In the PHATE method, users can select number of neighbors and principal components. In the correlation option, users can select Spearman or Pearson correlations.

For clustering, several methods were employed, including K-means, Gaussian Mixture Models (GMM) [48], Agglomerative Clustering, Self-Organizing Maps (SOM) [49], and Hierarchical Density-Based Spatial Clustering of Applications with Noise (HDBSCAN) [50], using scikit-learn and MiniSom. On the SurvSig website, users can also modify advanced parameters for the clustering methods. In case of k-means, apart from number of clusters, users can modify the initialization method, relative tolerance, max iterations and k-means algorithm. During Gaussian Mixture Models, users can select initialization method, covariance type, convergence threshold and non-negative regularization. In agglomerative clustering, users can select the linkage method. In DynamicTreeCut, users can select the linkage methods, distance metric and minimum cluster size. In HDBSCAN, users can select the alpha value, metric method, minimum number of samples, epsilon values and cluster selection method. In the OPTICS approach, users can select the minimum number of samples, distance metric, initialization method and epsilon value. Together, these options enable users to modify and refine both the dimensionality reductions and clustering approaches.

Correlations between gene expression profiles were calculated using Spearman or Pearson methods, implemented via pandas (versions 2.2.2 and 2.2.3).

### 4.4 Gene set enrichment, survival and pathway enrichment analysis

Gene set enrichment analysis was conducted using single-sample GSEA (ssGSEA) with the *ssgsea* function of gseapy (1.1.3) [51] using default settings. Since datasets were log-normalized, normalization was set to “None” during analyses seen in the figures. We have implemented advanced settings for ssGSEA calculation, which includes the option to select normalization, correlation method and weights. Pathway enrichment analysis was performed using clusterProfiler (4.6.2) [52] with gene annotations from org.Hs.eg.db (3.16.0) and results visualized using enrichplot (1.18.4). On the SurvSig website, users can select the enrichment method of clusterProfiler: *enrichGO* (where BP, MF CC or all sub-ontologies can be selected), or *enrichKEGG.* In addition, we implemented additional options, such as p-value and Q-value selection, selection of p-value correction, selecting the maximum number of genes and pathways to be plotted and ordering of gene lists on plots.

Survival analysis used the survival R package (3.7–0) for Kaplan-Meier estimations and Cox proportional hazards models, with additional visualization provided by survminer, such as multivariate analysis (0.4.9). On the SurvSig website, the multivariate option for clustered heatmaps can be found on a separate tab (“Multivariate & Chi [2]), while for survival analysis using ssGSEA values or single-genes, multivariate analysis is performed automatically. In each case, we implemented options to modify the analysis, which includes selection of clinical features and annotations to include in the analysis, and what should be the reference category.

In case of single-gene and ssGSEA score-based survival analysis, we implemented several options to stratify patients to “high” and “low” groups: 1) “Median” value; 2) “Percentage decomposition”: percentile based separation, where user can set a manual percentage value; 3) “Percentage decomposition (lower and upper limit)”: users can specify two percentages, where the “low” group consists of patients under the lower threshold, and “high” group consists of patients above the higher threshold; 4) “Automatic”: in this case, SurvSig scans by default all cutoffs in a 1 % step between 10 % and 90 % interval of patients, returning the percentage where the survival analysis resulted in the lowest p-value. To control for multiple testing, in these cases an additional plot and table appears summarizing the calculated p-values and adjusted (FDR) p-values. Both the interval range, and step size can be adjusted by the users. 5) “Expression values cutoff”: users can also select what expression value to use to separate the patients.

Heatmaps were generated using ComplexHeatmap (2.14.0) [53] to visualize gene expression and enrichment data. On the SurvSig website, users can choose to visualize (and cluster) data based on Z-scoring, or simply the normalized (log transformed in case of RNA-seq) expression data. We implemented several options to customize the heatmaps, such as specifying the color palett (and percentile thresholds), displaying dendograms of rows and columns and selecting annotations of samples together with their colors.

### 4.5 Statistical analyses

Comparisons and statistical analyses were calculated using SurvSig. Comparisons between groups (such as boxplots) were calculated using the *mannwhitneyu*() function of SciPy in python. Spearman correlations between genes (and ssGSEA enrichment scores) are calculated using the *spearmanr* function from SciPy, with implemented options to calculate Pearson correlation using the *pearsonr* function of SciPy.

For statistical analysis, false discovery rate (FDR) adjustments, chi-square tests, and Z-score calculations were performed using statsmodels (0.14.4), while median, standard deviation, and variance were calculated using numpy. Correlations between clinical and gene expression data were calculated using pandas. Interactive visualizations were generated using Plotly (5.23.0).

### 4.6 Gene set finder

The Gene Set Finder identifies significant genes using machine learning and statistical methods. Dimensionality reduction techniques, such as PCA, ICA, FA, and NMF, are applied to reduce complexity while retaining relevant structure. These methods, implemented via scikit-learn, were supplemented with standard deviation to rank genes by variability. For clinically integrated analysis, classifiers such as Artificial Neural Networks (ANN) and Categorical Naive Bayes were used to identify genes associated with clinical features. Kruskal-Wallis tests, corrected for multiple testing using FDR, were used to detect associations between genes and clinical characteristics.

## CRediT authorship contribution statement

**Gabriella Mihalekné Fűr:** Writing – review & editing, Writing – original draft, Visualization, Investigation. **Alexandra Benő:** Writing – review & editing, Writing – original draft, Visualization, Investigation. **Schultz Christopher W:** Writing – review & editing, Writing – original draft, Methodology, Investigation, Formal analysis. **Petronella Topolcsányi:** Writing – review & editing, Writing – original draft, Visualization, Investigation. **Éva Magó:** Writing – review & editing, Writing – original draft, Visualization, Investigation. **Parth Desai:** Writing – review & editing, Writing – original draft, Formal analysis. **Nobuyuki Takahashi:** Writing – review & editing, Writing – original draft, Formal analysis. **Mirit I Aladjem:** Writing – review & editing, Writing – original draft, Investigation, Formal analysis. **William Reinhold:** Writing – review & editing, Writing – original draft, Investigation, Formal analysis. **Yves Pommier:** Writing – review & editing, Writing – original draft, Investigation, Formal analysis. **Anish Thomas:** Writing – review & editing, Writing – original draft, Formal analysis, Conceptualization. **Pongor Lorinc:** Writing – review & editing, Writing – original draft, Supervision, Software, Methodology, Formal analysis, Conceptualization. **Kolos Nemes:** Writing – review & editing, Writing – original draft, Software, Methodology, Data curation, Conceptualization.

## Code availability

The SurvSig website is freely available at https://survsig.hcemm.eu/. The code is available at GitHub (link https://github.com/HCEMM/SurvSig)

## Declaration of generative AI and AI-assisted technologies in the writing process

During the preparation of this work the author(s) used OpenAI ChatGPT (4o) in order to proofread the text. After using this tool/service, the author(s) reviewed and edited the content as needed and take(s) full responsibility for the content of the publication.

## Funding

This research was funded by the János Bolyai Research Scholarship of the Hungarian Academy of Sciences BO/00697/23 (L.S.P). The project received funding from the EU's Horizon 2020 Research and Innovation Program with grant agreement No. 739593. Project no. TKP-2021-EGA-05 and 2022–2.1.1-NL-2022–00005 has been implemented with the support provided by the Ministry of Culture and Innovation of Hungary from the National Research, Development and Innovation Fund, funded by the TKP2021-EGA and National Laboratories grant program. The funders had no role in the study design, data collection and analysis, decision to publish, or preparation of the manuscript. Funders have no conflict of interest.

## Declaration of Competing Interest

The authors declare that they have no known competing financial interests or personal relationships that could have appeared to influence the work reported in this paper.
