# Supplementary material for: SurvSig: Harnessing gene expression signatures to uncover heterogeneity in lung neuroendocrine neoplasms
Source: Comput Struct Biotechnol J. 2025 Jun 6;27:2574–83. doi: 10.1016/j.csbj.2025.06.010 (PMC12205313; doi:10.1016/j.csbj.2025.06.010)
Supplement: Supplementary file 1 — Supplementary material [file mmc1.docx]

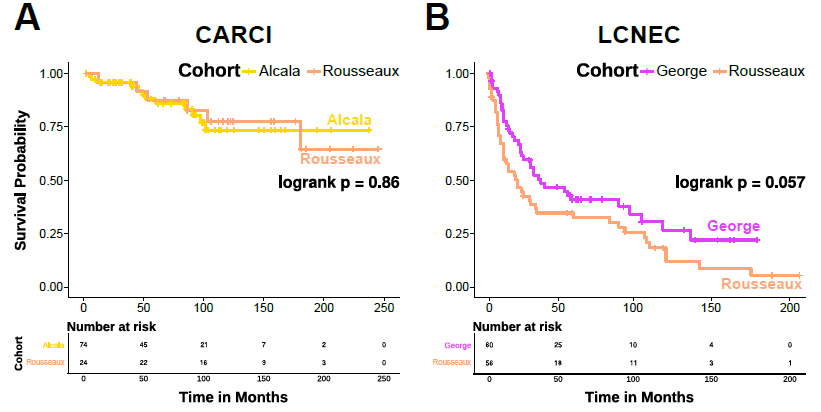


**Figure S1.** *Kaplan-Meier survival comparison of A) carcinoid patients and B) LCNEC patients from two cohorts.*


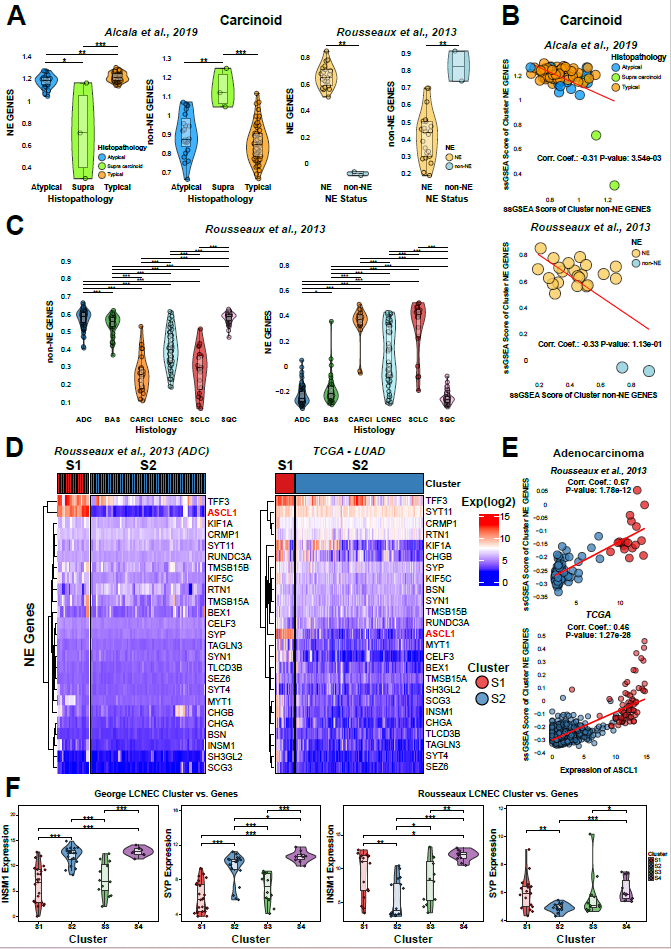


***Figure S2.*** *A Distribution of NE and non-NE gene set enrichments in the Alcala cohort (left) and Rousseaux* carcinoids (right)*. B) Spearman correlation between the NE and non-NE geneset activities in carcinoid samples from the Alcala (top) and Rousseaux (bottom) cohorts. C) Distribution of non-NE (left) and NE (right) geneset activities in all major histologies of the Rousseaux cohort. D) Heatmap of NE genes in the Rousseaux ADC adenocarcinoma (left) and TCGA adenocarcinoma (right) cohorts. Patients grouped into ASCL1 high and low cases. E) Spearman correlation of ASCL1 expression and NE geneset score in adenocarcinoma tumors from the Rousseaux (top) and TCGA adenocarcinoma (bottom) cohorts. F) Comparison of INSM1 and SYP expression in the identified LCNEC patient clusters in the George LCNEC cohort (left) and Rousseaux cohort LCNEC samples (right). Distributions of sample groups seen in panels A,C and F were compared using Mann-Whitney U tests (where p-values are shown as *: p < 0.05, **: p < 0.01, ***: p < 0.001).*

*.*


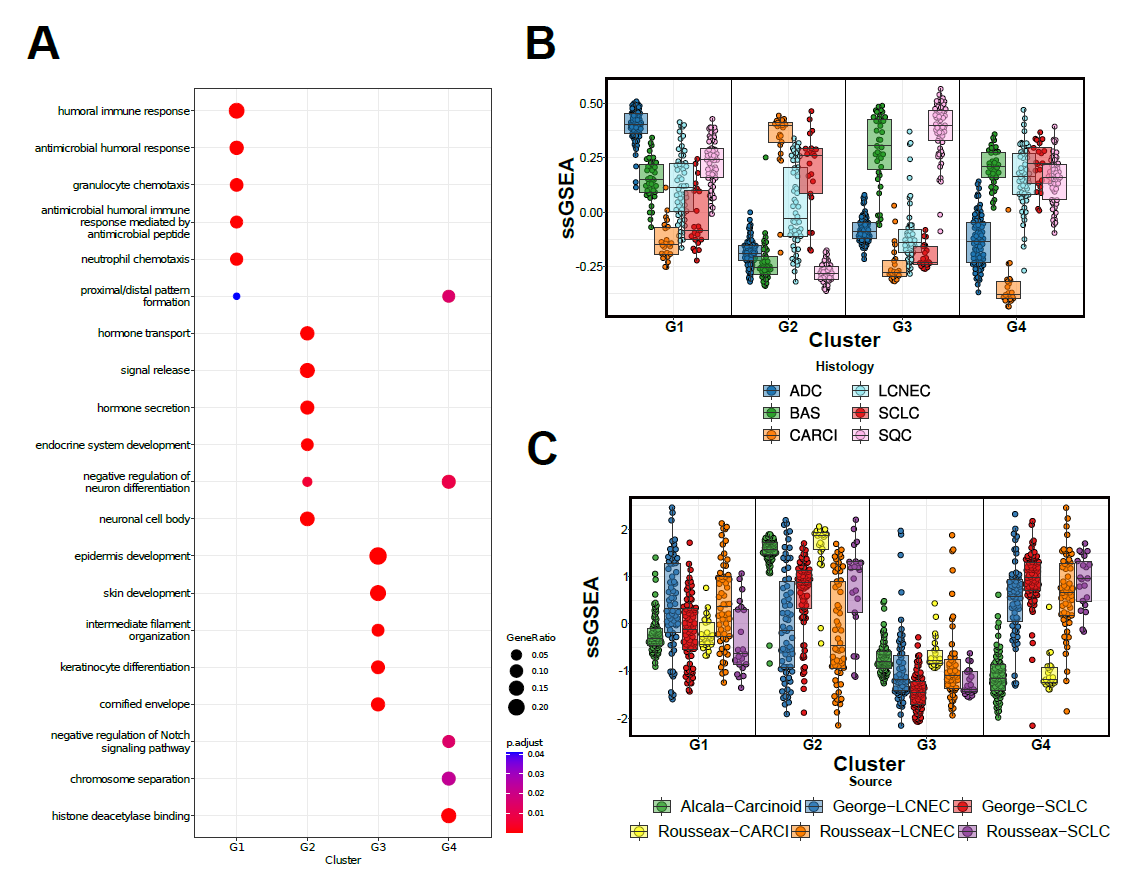


***Figure S3.*** *A) Gene ontology analysis results of gene clusters (related to figure 3). B) ssGSEA enrichment distribution of gene sets across all histologies or the Rousseaux cohort. C) Raw enrichment scores of NE specific genesets from multiple data sources.*

***
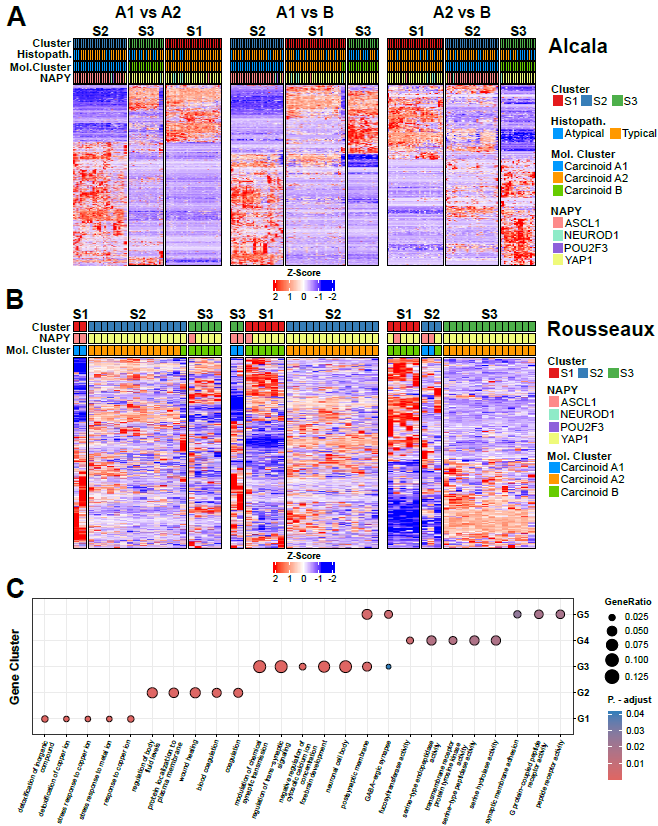
Figure S4.*** *A-B) Expression heatmaps using the three gene lists that defined molecular subtypes of carcinoids using the A) Alcala and B) Rousseaux carcinoid cohorts. C) Gene ontology results for gene clusters in the newly defined gene signature that stratifies molecular subtypes of carcinoids (related to Figure4 A-B).*


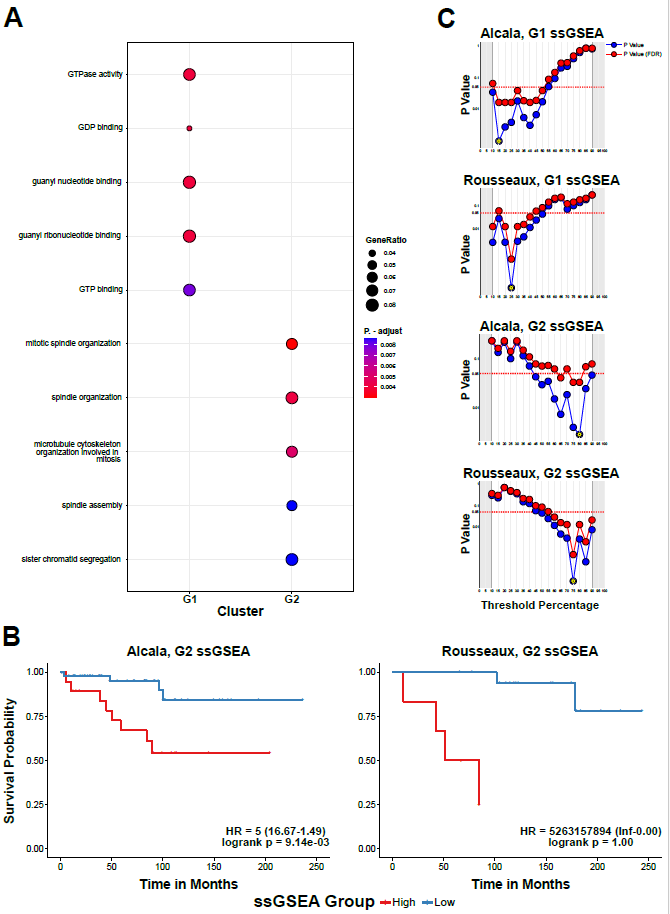


**Figure S5.** A) Gene ontology results of typical (G1) and atypical (G2) enriched genes, related to Figure 4C. B) *Kaplan-Meier survival plots of high and low G2 gene set activities. C) Automatic cutoff selection for the ssGSEA-based survival analysis results in significant results after p-value adjustment.*
